# Does bimanual coordination training benefit inhibitory function in older adults?

**DOI:** 10.3389/fnagi.2023.1124109

**Published:** 2023-04-06

**Authors:** Marta Maria Torre, Antoine Langeard, Louis Alliou, Jean-Jacques Temprado

**Affiliations:** ^1^Aix-Marseille Université and CNRS, ISM UMR 7287, Institut des Sciences du Mouvement, Marseille, France; ^2^Normandie Université, Université de Caen Normandie, Institut National de la Santé et de la Recherche Médicale, COMETE, Caen, France

**Keywords:** bimanual coordination training aging, cognition, inhibition function, cognitive-motor training, bimanual coordination

## Abstract

**Introduction:**

Whether complex movement training benefits inhibitory functions and transfers the effects to non-practiced motor and cognitive tasks is still unknown. The present experiment addressed this issue using a bimanual coordination paradigm. The main hypothesis was that bimanual coordination training allows for improving the involved cognitive (i.e., inhibition) mechanisms and then, transferring to non-practiced cognitive and motor tasks, that share common processes.

**Methods:**

17 older participants (72.1 ± 4.0 years) underwent 2 training and 3 test sessions (pre, post, and retention one week after) over three weeks. Training included maintaining bimanual coordination anti-phase pattern (AP) at high frequency while inhibiting the in-phase pattern (IP). During the test sessions, participants performed two bimanual coordination tasks and two cognitive tasks involving inhibition mechanisms. Transfer benefits of training on reaction time (RT), and total switching time (TST) were measured. In the cognitive tasks (i.e., the Colour Word Stroop Task (CWST) and the Motor and Perceptual Inhibition Test (MAPIT)), transfer effects were measured on response times and error rates. Repeated one-way measures ANOVAs and mediation analyses were conducted.

**Results:**

Results confirmed that training was effective on the trained task and delayed the spontaneous transition frequency. Moreover, it transferred the benefits to untrained bimanual coordination and cognitive tasks that also involve inhibition functions. Mediation analyses confirmed that the improvement of inhibitory functions mediated the transfer of training in both the motor and cognitive tasks.

**Discussion:**

This study confirmed that bimanual coordination practice can transfer training benefits to non-practiced cognitive and motor tasks since presumably they all share the same cognitive processes.

## 1. Introduction

As we age, cognitive functions undergo a decline that is often considered a forerunner of neurodegeneration and loss of behavioral adaptability in everyday tasks. It is now widely admitted that these alterations can be attenuated or delayed in healthy older adults by cognitively and physically enriched life habits. Therefore, understanding how effective training protocols can prevent cognitive decline in healthy older adults is a major challenge for the aging research community (Erickson et al., 2013; Voss and Jain, 2022).

For a long time, age-related alterations in cognitive and motor domains have been considered separately (Zapparoli and Mariano, 2022). Accordingly, cognitive training was hypothesized to be the only means to improve cognitive functioning. By demonstrating the benefits of aerobic exercise on cognition, Colcombe and Kramer (2003) played a pivoting role in the Copernican revolution that led to considering physical exercises as a critical means to enhance brain functions and cognitive performance. Since then, several studies have confirmed the benefits of endurance and muscular resistance training on executive functions, attention and memory (e.g., Netz, 2019). More recently, it has been reported that older individuals that have a high level of motor fitness (Voelcker-Rehage, 2008; Voelcker-Rehage et al., 2010; Ludyga et al., 2020) or who participated in complex coordination training programs demonstrated superior cognitive performance, especially in executive functions and perceptual speed (Voelcker-Rehage et al., 2011; Niemann et al., 2014). These findings suggested that repetitive practice of complex movements might be a very effective strategy to improve brain functions and cognition in older adults due to embodiement of cognition in human behavior (e.g., Raab and Araújo, 2019). It might be the case since, due to the so-called age-related cognitive-motor dedifferentiation, cognitive mechanisms and their related brain structures become more and more involved in movement control (e.g., Sleimen-Malkoun et al., 2013). However, while there is abundant literature on the effects of endurance or muscular resistance training on cognitive performance interventional studies that have investigated whether and how complex movement training may transfer to the efficiency of cognitive functions in older adults are scarce. The present experiment addressed this issue by using a rhythmic bimanual coordination paradigm.

In a laboratory context, bimanual coordination is frequently characterized by two stable and flexible patterns [in-phase (IP) and anti-phase (AP)] (Kelso, 1984). Conventionally, the in-phase (IP) pattern is achieved through the simultaneous activation of homologous forearm muscles groups thereby giving rise to mirror-symmetrical movements concerning the body midline; while the anti-phase pattern (AP) is achieved through the simultaneous activation of non-homologous muscles groups thereby one limb moves toward the body midline, while the other limb moves away from it and vice versa (e.g., Temprado et al., 2010, 2020). AP and IP coordination patterns can be captured by the value of relative phase (RP) between the two hands (180° and 0°, respectively), while their stability can be indexed by the magnitude of fluctuations of RP (i.e., the SD of RP) (Kelso, 1984; Haken et al., 1985; Temprado et al., 2010). The dynamics of bimanual coordination reflect i) the existence of these stable patterns and ii) the appearance of spontaneous transitions from AP to IP when the frequency of movements increases.

Over the last 20 years, the role of cognition in the control of bimanual coordination patterns has been the subject of numerous studies in young (Lee et al., 1996; Pellecchia and Turvey, 2001; Temprado et al., 2002, 2010; Pellecchia et al., 2005; Shockley and Turvey, 2005) and older adults (Wishart et al., 2000; Lee et al., 2002; Temprado et al., 2010, 2020). In particular, it has been shown that attention and/or inhibition mechanisms were involved in bimanual coordination (e.g., Fujiyama et al., 2009; Levin et al., 2014), in the voluntary stabilization of existing patterns (Monno et al., 2002; Temprado et al., 2010), in the inhibition of spontaneous transitions (Lee et al., 1996; Temprado et al., 2002), and in the voluntary transition between the AP and IP patterns (Temprado et al., 2020). These findings open the door to the development of training protocols, grounded on bimanual coordination tasks, to improve the efficiency of cognitive functioning in older adults, and especially, inhibition functions, which are highly affected by age-related decline. In the present study, we capitalized on this framework to investigate whether, in healthy older adults, bimanual coordination training in conditions that required strong involvement of inhibition processes transferred to non-practiced cognitive and motor tasks also involving inhibitory mechanisms.

To verify this hypothesis, we assessed the effects of bimanual coordination training on the ability to maintain the AP pattern at high oscillations frequencies that is, to inhibit spontaneous transition to the IP pattern. According to a previous study (Temprado et al., 2002), we expected to observe that training delayed the transition frequency at which the spontaneous transition occurred. Then, we assessed the consequences of bimanual coordination training on performance in a non-practiced intentional pattern switching task (AP to IP et IP to AP), similar to those previously used by Temprado et al. (2020). To fulfill this objective, we assessed transfer effects of training in post-test and retention test conditions carried out immediately and 1 week after the training session, respectively. Consistent with a previous study (Temprado et al., 2002), the retention test should allow to determine whether an overcompensation of training effects take place during the retention period.

We expected to observe a decrease in switching times, especially for the AP to IP direction, as a result of bimanual training. Finally, we tested the effects of bimanual training in two non-practiced cognitive tasks that is, the Color Word Stroop Test and the MAPIT. We expected to observe decreases in response times, in the two tasks. Such results would demonstrate the existence of transfer effects, presumably due to the training of common (inhibition) processes between the bimanual coordination task and the non-practiced cognitive tasks. To further understand the transfer effects, if existed, we used mediation analyses to determine whether performance improvement in the non-practiced tasks resulted from the mediation of inhibition mechanisms trained during bimanual coordination practice.

## 2. Methods

### 2.1. Participants

Only older adults were tested. Seventeen participants, 6 women and 11 men (mean age 72.18 ± 4.04), were included according to the following self-reported criteria: (i) age ≥ 65 ≤ 80 years, (ii) normal or corrected-to-normal vision and hearing, and (iii) agreeing to follow the entire protocol (5 participants weren't able to finish the program). The non-inclusion criteria were: (i) pain or disability affecting the hand, arm, or shoulder (e.g. arthritis), (ii) upper or lower limb surgery in the last 6 months, (iii) an Mini Mental State Ewamination (MMSE) score ≤ 24 (Trzepacz et al., 2015) (mean 28.8 ± 1.2). Participants signed written informed consent.

### 2.2. Design

The protocol was approved by the French ethic committee CERSTAPS IRB00012476-2022-12-05-181. It consisted of two training sessions in a bimanual coordination task and three testing sessions (pre-test, post-test, and retention) in two bimanual coordination tasks and two cognitive tasks. In each testing session, both training and transfer effects were assessed. Training effects were assessed in the practiced bimanual coordination task while transfer effects were assessed in the non-practiced motor and cognitive tasks. The 5 sessions (of about 75 min each) were spread over 3 weeks. During the first week, participants completed the pre-test. During the second week, they performed two training sessions and one post-test session on three different days (i.e., with 1 day off in between). During the third week, 7 days after the second training session, they performed the retention test (see Table 1). For the evaluation session, the order of presentation of cognitive and motor tests were randomized. Exception was made for the spontantaneous transition frequency test, which served as assessment test and, accordingly, was always performed before the others.

**Table 1:** Overview of the protocol.

	**First week**	**Second week**			**Third week**
	**Pre-test**	**First training session**	**Second training session**	**Post-test**	**Retention-test**
Cognitive test	MMSE Stroop test MAPIT test	50 trials of AP at increasing frequency	50 trials of AP at increasing frequency	Stroop test MAPIT test	Stroop test MAPIT test
Bimanual coordination tests	Spontaneous transition frequency detection test (10 trials) Intentional switching: 10 trials in each direction (IPtoAP and APtoIP)			Spontaneous transition frequency detection test (10 trials) Intentional switching: 10 trials in each direction (IPtoAP and APtoIP)	Spontaneous transition frequency detection test (2 x 10 trials) Intentional switching: 10 trials in each direction (IPtoAP and APtoIP)

### 2.3. Intervention

**Figure 1:**
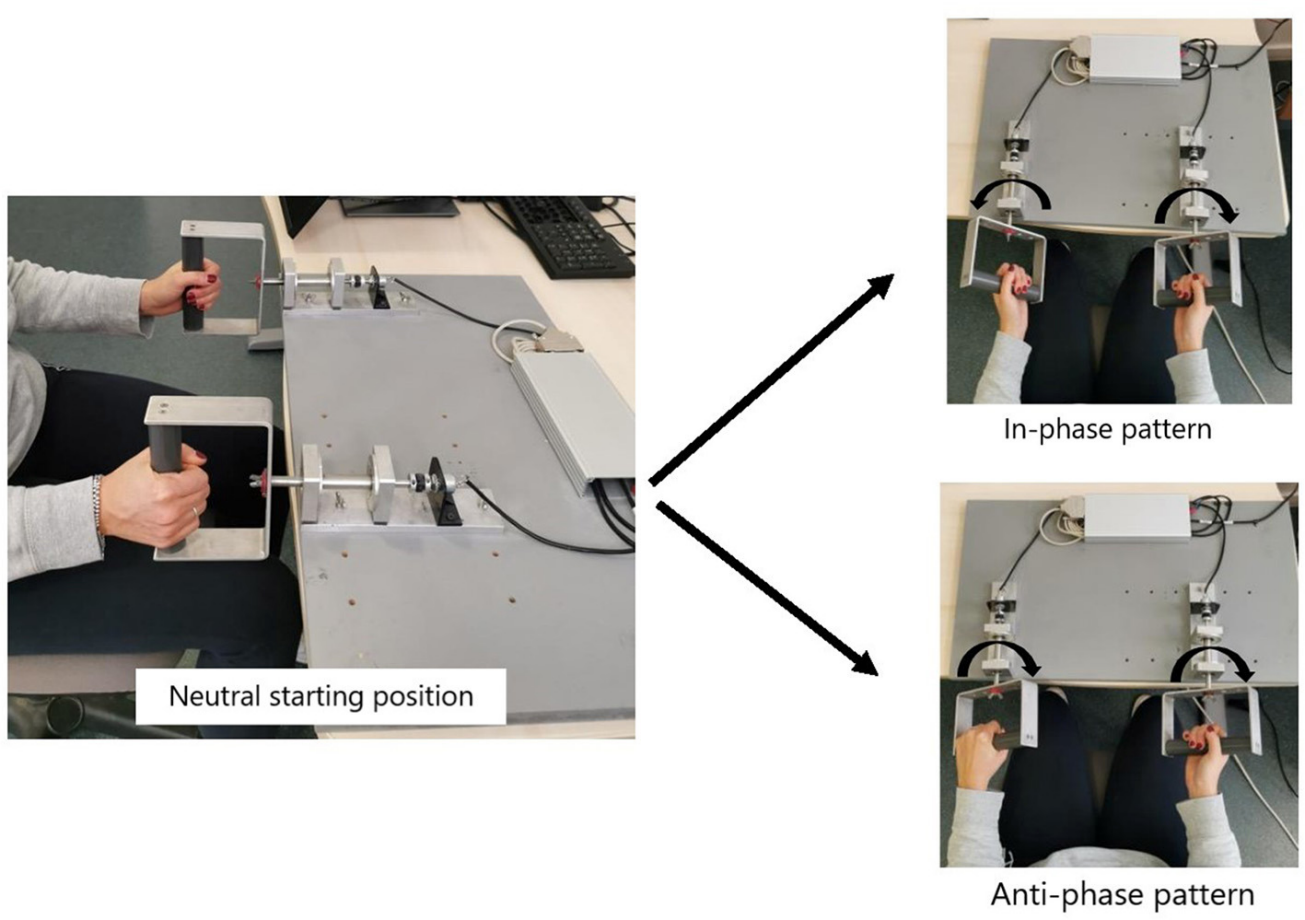
Device used for the bimanual coordination task and visual representation of IP (In-phase) and AP (Anti-Phase) patterns.

Participants had to perform the AP pattern, starting at the transition frequency (TF) identified in the pre-test, minus 0.25 Hz (see below). The training frequency was then increased gradually by 0.25 Hz after each block of 10 trials. They were instructed to resist to the transition to IP that is, to inhibit the switching from AP to IP (see description of patterns in Figure 1). Bimanual coordination training was divided in two separate sessions of 50 trials each (i.e., a total of 100 trials of 20 s).

### 2.4. Testing

Two motor and two cognitive tasks were used during the testing sessions. The effect of bimanual coordination training was assessed through the measurement of changes in the spontaneous transition frequency (TF), while transfer effects were assessed through performance measurement in (unpracticed) intentional switching motor task, Stroop task and the MAPIT, respectively.

#### 2.4.1. Spontaneous transition frequency

The spontaneous transition frequency (TF) between the AP and IP patterns was measured in each testing session. The bimanual coordination task consisted of pronation-supination movements of the forearms in the frontal plane, in synchronization with a metronome according to the AP or IP pattern. For each of the two coordination patterns, 3 to 5 familiarization trials, consisting of oscillating at their spontaneous frequency, were provided to participants. Then, the transition frequency was determined by asking participants to perform the AP pattern, following an auditory metronome. The oscillation frequency was increased from 1 to 3 Hz by steps of 0.25 Hz, changing every 10 s. They were instructed not to resist when they felt they were losing the AP coordination for the easier IP pattern. Ten trials were performed. The effective frequency at which transition occurred to the IP pattern (which may be different from the frequency prescribed by the metronome) was recorded online by a lab-customized LabVIEW program. The effective frequency at which a transition was observed for at least 60% of the trials was considered the reference frequency for the training session. Effective oscillation frequencies were calculated based on averaged cycle periods in each metronome condition. Mean values of discrete relative phase (DRP) allowed us to determine when the transition started.

The procedure used to analyze bimanual coordination performance was similar to the one used by Temprado et al. (2020). The raw signals were processed with two customized Matlab routines (MathWorks Inc, Natick, M.A, United States). The first 5 s of each trial allowed to ensure that a stable pattern was performed. They were filtered with a Butterworth filter (cut-off frequency 10 Hz, order 2). Then, the amplitude centering procedure was used to remove frequency artifacts of the non-sinusoidal signals, when existing. According to Lamb and Stöckl (2014), the normalization was based on the function:


$$\begin{array}{l} \left(y\left(ti\right)\right)=2\left(\frac{2\left(y\left(ti\right)-\min\left(y\left(t\right)\right)\right)}{\max\left(yt\right)-\min\left(y\left(t\right)\right)}\right)-1 \end{array}$$

This function transformed the original values y(t) in such a way that the minimum value of g(y(t)) equals −1 and the maximum value of g(y(t)) equals 1.

*Detection of spontaneous transition frequency*. Effective oscillation frequencies were calculated on the basis of averaged cycle periods in each metronome conditions. Then, mean values and SD of discrete relative phase (DRP) were calculated and tracked to determine when the transition started. The transition was considered starting when the last value of DRP = 180° ± 45° was followed by five consecutive DRP values lower than 135°. The same procedure was applied to identify the end of transition to the IP pattern. The transition considered accomplished when the value of DRP post-transition was equal to 0°±45° for at least 3 consecutive cycles. Notably, a transition occurring during the first second of a given step of frequency was considered occurring in the previous step.

#### 2.4.2. Intentional switching between bimanual coordination patterns

Performance in intentional switching tasks was assessed, in two directions (APtoIP and IPtoAP) to determine whether the effects of bimanual coordination training transferred to an unpracticed motor task presumably involving similar inhibition mechanisms. Two blocks of 10 trials from AP to IP and from IP to AP were presented in random order. The oscillation frequency was paced by a metronome set at the transition frequency (TF) identified in the pre-test, minus 0.25 Hz. Between the 15th and 18th s, the metronome was changing its signal's tone, indicating to the participants to change as fast as possible from the ongoing pattern to the other one. Each trial lasted 30 s.

According to our previous study (Temprado et al., 2020), to analyze intentional switching, in each condition, we calculated the continuous relative phase (CRP), after the application of the Hilbert transform sign according to the following formula:


$$\begin{array}{l} CRP\;\left(t_{i\;}\right)=\;CRP_{left}\left(t_{i\;}\right)\;CRP_{right}\left(t_{i\;}\right)\;\\ \;\arctan\left(\frac{H_{1}\left(t_{1}\right)x_{2}\left(t_{1}\right)-\;H_{2}\left(t_{1}\right)x_{1}\left(t_{1}\right)}{x_{1}\left(t_{1}\right)x_{2}\left(t_{1}\right)-\;H_{21}\left(t_{1}\right)H_{2}\left(t_{1}\right)}\right) \end{array}$$

Then, we calculated the mean and SD of the CRP for each participant. After calculation, the times series of CRP were divided into pre-switching and post switching phases (Lamb and Stöckl, 2014). For the pre-switching phase, we calculated the mean and SD of CRP. CRP artifact was taken into consideration, two cycles before and one after were deleted, so we calculated the CRP two cycles before and one after our region of interest. The TST was defined as the time lapsing between the switching signal and the first mean value of CRP post-transition that preceded at least 3 s of stabilization within a range of 45° around the CRP value corresponding to the requested pattern (i.e., either IP or AP). In addition, the switching phase was decomposed into RT that is, the interval between the signal of changing given by the metronome and the first value of RP outside of +/– 45° of the value corresponding to the currently performed pattern (i.e., 180° or 0°).

**Figure 2:**
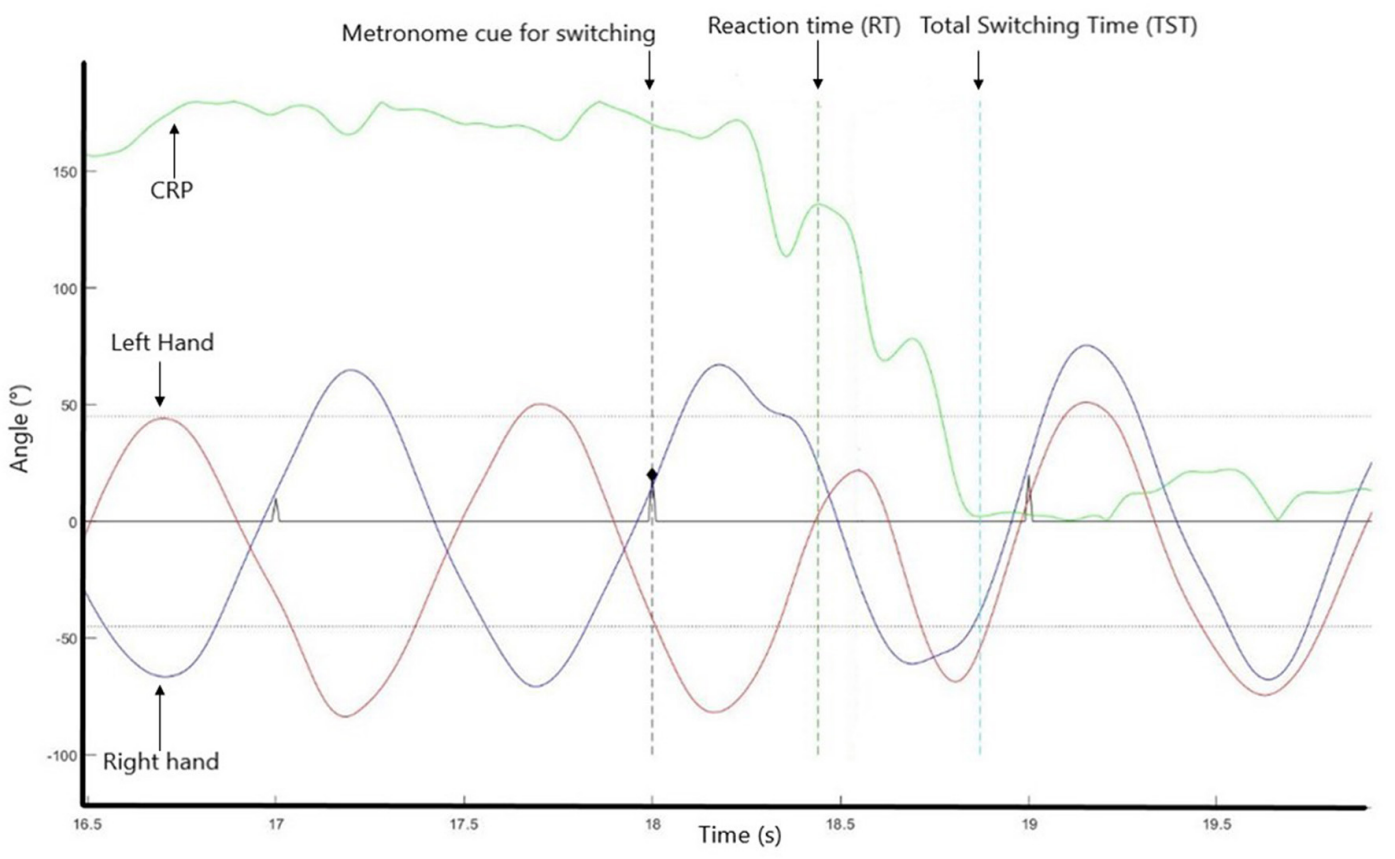
Decomposition of the switching phase into different sub-parts.

Reaction times (RT) and Total Switching Times (TST) were calculated for the two switching directions. In each condition, continuous relative phase (CRP) was calculated for each participant. Then, the times series of CRP were divided into pre-switching and post-switching phases (Lamb and Stöckl, 2014). For the pre-switching phase, two measures of response times were defined (see Temprado et al., 2020, for a similar procedure). The TST was the time lapse between the switching signal and the first mean value of CRP post-transition that preceded at least 3s of stabilization within a range of 45° around the CRP value corresponding to the requested pattern (i.e., either IP or AP). The RT was the interval between the signal of change given by the metronome and the first value of RP outside of +/– 45° of the value corresponding to the currently performed pattern (i.e., 180° or 0°) (see Figure 2 for visual details).

#### 2.4.3. Cognitive and motor inhibition

The Color Words Stroop Test (CWST) was used to test cognitive inhibition. It was carried out on a computer with lab-customized software. Participants were comfortably seated in front of a screen (Dell24 P2418HT, 23.8 inches). A colored word appeared on the screen and they were asked to indicate the color of the word by pressing the corresponding letter on the keyboard in front of them as quickly as possible. The keyboard was adapted so that only the letters required for the test were visible on the keyboard. Four different colors were used: green, blue, red, and yellow. Thus, depending on the consistency between semantics and color, the condition was considered either congruent (C) or incongruent (I). Neutral trials (N) were also presented in different words (e.g., arm, leg...) and were written in one of the different colors (green, blue, red, and yellow). After familiarization with 9 words not used for the test, 75 trials were presented randomly for testing (25 congruent, 25 incongruent, and 25 neutral; color words and answers per color were balanced). Each word remained on the screen until the answer was given. In each condition, the number of errors and the reaction time (RT) that is, the time elapsed between the appearance of the word and the pressing of the key on the keyboard, were recorded. Using a similar procedure as those used by Temprado et al. (2020), we didn't calculate inhibition costs.

**Figure 3:**
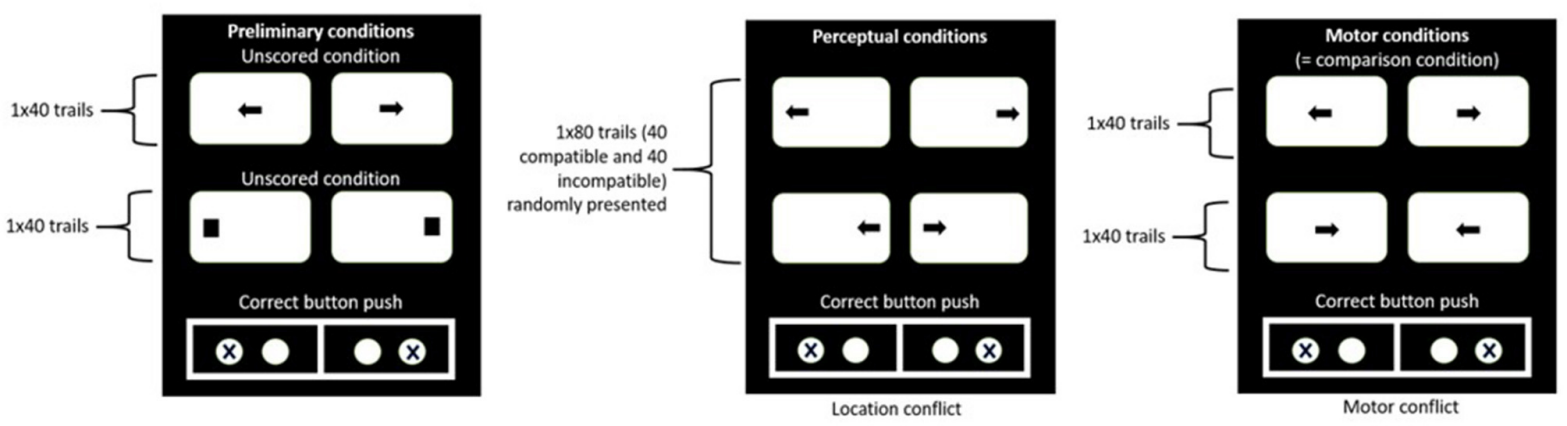
Panels of stimuli presented in the different conditions of the MAPIT.

The MAPIT (Nassauer and Halperin, 2003; Jennings et al., 2011) was used to assess, separately, perceptual (PI) and motor inhibition (MI). The test was carried out on a computer lab-customized software (ICE ^®^ software, https://trello.com/b/EtNCNrZH/ice). Its general principle consisted of responding as fast as possible to the direction or location of arrows presented on the screen (Dell24 P2418HT, 23.8 inches) by pressing a corresponding key on a modified keyboard, in which only two keys (“Q” and “M”), used for the right direction and left directions, respectively, were visible (see Figure 3). Each trial started with the fixation of a black cross presented in the center of the screen, which disappeared when the arrow appeared and remained on the screen until the participant pressed the key. The test consisted of 3 different blocks of trials designed to assess either perceptual or motor inhibition (see Figure 3): (i) *a preliminary block* of 80 trials used as familiarization in which participants had to press the key corresponding to the direction an arrow or square's location on the screen (i.e., either right or left), (ii) *a perceptual inhibition block* of 80 trials in which participants had to press the button corresponding to the direction of the arrow's pointing, even if the location of the arrow was opposite (e:g. arrow pointing to the left and placed on the right of the screen), and (iii) *a Motor inhibition block* of 80 trials in which the arrows were presented in the center of the screen and participants were asked to either to press the button corresponding to the direction of the arrow's pointing, or the opposite direction (for details see supplementary material). Based on measured median reaction times (RT) (Jennings et al., 2011), perceptual (PI) and motor (MI) interference scores were calculated as follows:

PI = Median RT of the perceptual incongruent – Median RT of the perceptual congruent condition

MI = Median RT of the motor incongruent condition – Median RT of the motor congruent condition.

### 2.5. Statistical analyses

One-way repeated-measures ANOVAs (SPSS Inc., Chicago, IL, USA) were used to compare the performance measured during pre-test, post-test, and retention test in: (i) the trained bimanual coordination task (transition frequency, TF). A two-ways ANOVA was used to test the effects of time (test) and direction (APtoIP/IPtoAP) on switching times in the bimanual switching task, while two ways (condition x time) ANOVAs were used to compare the performance observed in the CWST (RT of neutral, congruent, and incongruent conditions) and in the MAPIT (for PI and MI interference scores). The Shapiro-Wilk Normality test was performed before all, and Newman—Keuls *post hoc* analysis was also run.

In addition, mediation analyses were carried out following the procedures from mediation and moderation in repeated measures design, using the MEMORE macro for IBM SPSS Statistics (IBM Corp., Armonk, NY, United States) (Montoya and Hayes, 2017). Mediation analysis allows to quantify the degree to which a mediator (M) acts as the “mechanism” by which an independent factor (X) affects an outcome (Y). In this variant of mediation analysis, X does not actually exist in the data and represents the effect of the intervention influencing M and Y over time. The effects of bimanual coordination training (X) on Y corresponded to the total effects “c”, and the effects of the training on Y while controlling for the mediator are called the direct effects “c”. The effects of the training on M corresponded to the “a” path and the effects of M on Y to the “b” paths. The amount of mediation, the indirect effect (“ab”), refers to the role of the mediator (M) in the effect of the training on Y. We tested the significance of the indirect effects using bias-corrected bootstrap confidence intervals (CIs) (based on 2000 bootstrap samples). CI that did not contain zero represents significant effects, and therefore significant mediation of X on Y through M. The centrality and normality of the residuals were verified.

**Figure 4:**
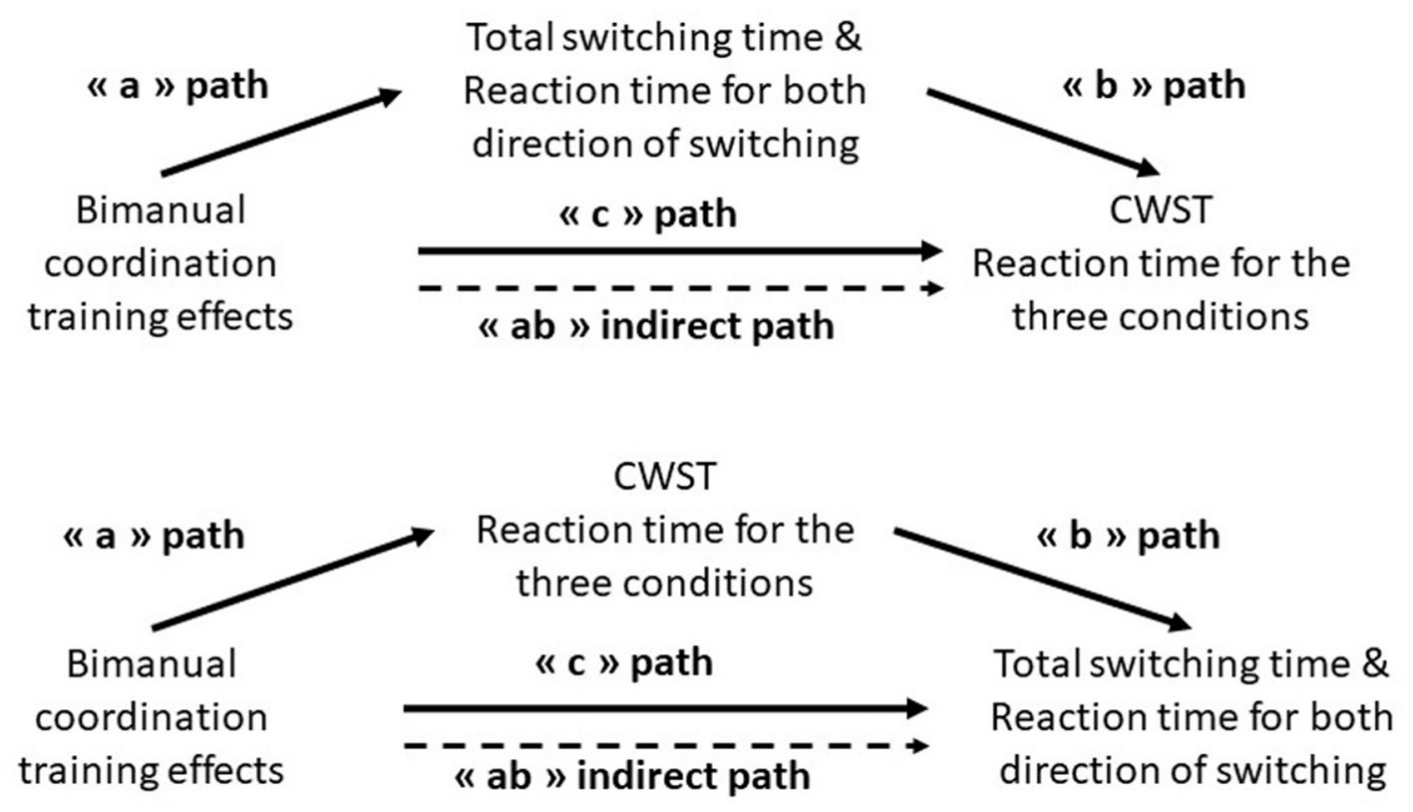
Illustration of the two mediation models.

Two sets of mediation analysis were carried out. The first one aimed to determine whether training-related enhancement of intentional switching variables (i.e., RT and TST) was mediated by improvements in inhibition capacities, as measured by the CSWT and the MAPIT. The second set of analyses aimed to determine whether transfer effects observed in CWST and MAPIT were mediated by enhanced performance in the intentional switching task. The threshold chosen for statistical significance is *p* < 0.05 (details in Figure 4). This type of mediations analysis has been recently widely used in a variety of research domains related to interventions, and in different populations (Bell et al., 2018; Boidin et al., 2020; Sidhu and Cooke, 2021).

## 3. Results

### 3.1. Training effects on the maintenance of the AP pattern

The one-way ANOVA performed on the mean effective transition frequency revealed an effect of time [*F*_(2, 32)_ = 69.83, *p* < 0.01]. For testing sessions, he Newman-Keuls *post-hoc* test revealed a significant improvement between the pre-test (1.68 ± 0.28) and the post-test (2.15 ± 0.32), as well as between the pre-test and the retention test (2.16 ± 0.37), of about 0.5 Hz, on average. In other words, transition frequency was significantly delayed after bimanual coordination training.

### 3.2. Transfer to the untrained intentional switching task

For TST, the two-ways ANOVA revealed significant main effects of direction [*F*_(1, 31)_ = 49.20, *p* < 0.01] and time [*F*_(2, 62)_ = 20.49, *p* < 0.01]. Specifically, the Newman-Keuls *post-hoc* test carried out on the two directions of switching revealed that TST was longer for the IP to AP direction than for the AP to IP direction (1,668 ± 525.04 ms, and 865 ±248.2 ms, (*p* < 0.00). Moreover, the post hoc analysis carried out on testing sessions revealed that independently of the switching direction, the TST observed in the pre-test was significantly longer than in the post-test and retention [1,505 ± 664.66 ms > 1,181 ± 530.79 and 1,151 ±474.27 ms; *p* < 0.001]. For RT, main effects of direction [*F*_(1, 31)_ = 5.59, *p* < 0.05] and time [*F*_(2, 62)_ = 3.74, *p* < 0.05] were observed. Specifically, the Newman-Keuls *post-hoc* test carried out on the two directions of switching revealed that RT was longer in the AP to IP direction than in the IP to AP direction (348 ± 83.65 ms, and 310 ± 59.92 ms; *p* < 0.02). Moreover, the *post-hoc* analysis carried out on testing sessions revealed that independent of the switching direction, RT was significantly longer during the pre-test than during the post-test and the retention test (358 ± 103.61 ms >319 ± 63.21 ms and 308 ± 67.97 ms; *p* < 0.03).

### 3.3. Transfer to the untrained cognitive tasks

For the CWST, the two-ways ANOVA revealed main effects of condition [*F*_(2, 48)_ = 3.28, *p* < 0.05] and time [*F*_(2, 96)_ = 47.30, *p* < 0.01] on response time. The incongruent condition was always slower than the congruent one, independent of the testing time (1,277 ms and 1,099 ms, respectively). Moreover, response times were shorter in the both the post-test and the retention test than in the pre-test (*p* < 0.05). Also, responses times were shorter in the retention test than in the post-test (pre-test: 1,272 ms; post-test: 1,168 ms; retention test: 1,090 ms). Interaction time x condition effect was not significant (*p* > 0.63).

For the MAPIT, the analysis revealed an effect of time on MI score [*F*_(2, 30)_ = 3.56, *p* < 0.05], which improved between the pre-test and the retention test (145 ms and 86 ms, respectively). A tendency (*p* = 0.07) was observed for the difference between the pre-test and the post-test 145 and 105 ms, respectively).

### 3.4. Mediation analyses

The results of the first set of mediation analyses are presented in Tables 2, 3. A significant indirect effect was found for the effect of the bimanual coordination training on the reaction time of the incongruent condition of the CWST, through the TST (52.5% of the total effect) and RT (23.8% of the total effect); in the AP to IP direction. The direct effect (≪ c' ≫) was not significant after taking into account the mediators, being consistent with a full mediation hypothesis (the training no longer affects Y after controlling for M). All other mediation analyses were inconsistent (negative ≪ ab ≫) or not significant. The results of the second set of mediation analyses are presented in Table 3. A significant indirect effect was found for the effect of the bimanual coordination training on the TST in the AP to IP direction, mediated by the RT observed in the incongruent condition of the CWST (39.0 % of the total effect). The direct effect (≪ c' ≫) was not significant after taking into account the mediators, being consistent with a full mediation hypothesis. All other mediation analyses were not significant. Mediation analyses carried out with the outcome variables of the MAPIT were not significant.

**Table 2:** Training-related enhancement of intentional switching variables (i.e., RT and TST) mediation through improvements in inhibition capacities as measured by the CSWT (unstandardized reported effect).

		**Coefficient**	**SE**	**t**	**P**
Relation ≪ a ≫	RT IP to AP	29.20	25.62	1.13	0.27
	TST IP to AP^*^	405.05	93.43	4.33	<0.01
	RT AP to IP	47.62	30.97	1.53	0.14
	TST AP to IP^*^	238.75	88.57	2.69	0.01
Relation ≪ c ≫ (Total Effect)	RT Congruent^*^	96.00	23.28	4.12	<0.01
	RT Neutral^*^	80.21	35.45	2.26	0.03
	RT Incongruent^*^	134.24	42.54	3.15	<0.01
		**Coefficient**	**BootSE**	**BootLLCI**	**BootULCI**
Bootstrap analyses of the indirect ≪ ab ≫ paths	**RT IP to AP**				
	RT Congruent	27.31	21.88	−0.8070	92.86
	RT Neutral	−5.90	23.18	−48.27	29.66
	RT Incongruent	16.74	31.23	−8.22	118.28
	**TST IP to AP**				
	RT Congruent	−10.96	29.38	−57.16	43.83
	RT Neutral	28.68	50.77	−34.76	148.89
	RT Incongruent^*^	−67.43	41.52	−165.52	−6.68
	**RT AP to IP**				
	RT Congruent	17.91	18.29	−3.77	69.53
	RT Neutral	6.67	23.11	−25.20	68.99
	RT Incongruent^*^	27.31	22.06	0.1147	103.58
	**TST AP to IP**				
	RT Congruent	34.93	36.85	−10.55	139.59
	RT Neutral	18.13	63.51	−142.34	148.59
	RT Incongruent^*^	60.21	87.36	11.54	321.00

**Table 3:** Training-related enhancement of inhibition capacities as measured by the CSWT mediation through improvements in intentional switching variables (i.e., RT and TST) (unstandardized reported effect).

		**Coefficient**	**SE**	**t**	**P**
Relation ≪ a ≫	RT Congruent^*^	96.00	23.28	4.12	<0.01
	RT Neutral^*^	80.21	35.45	2.26	0.03
	RT Incongruent^*^	134.24	42.54	3.15	<0.01
Relation ≪ c ≫ (Total Effect)	RT IP to AP	29.20	25.62	1.13	0.2712
	TST IP to AP^*^	405.05	93.43	4.33	<0.01
	RT AP to IP	47.62	30.97	1.53	0.14
	TST AP to IP^*^	238.75	88.57	2.69	0.01
		**Coefficient**	**BootSE**	**BootLLCI**	**BootULCI**
Bootstrap analyses of the indirect ≪ ab ≫ paths	**TST IP to AP**				
	RT Congruent	54.31	107.31	−277.17	155.16
	RT Neutral	18.48	58.40	−100.29	138.14
	RT Incongruent	−81.35	74.19	−277.76	14.02
	**TST AP to IP**				
	RT Congruent	77.60	69.15	−63.00	217.42
	RT Neutral	21.04	45.16	−37.96	131.32
	RT Incongruent^*^	93.17	65.44	18.22	297.41

## 4. Discussion

The present study aimed to determine if training in a bimanual coordination task involving inhibition mechanisms transferred to untrained motor and/or cognitive tasks involving similar mechanisms.

### 4.1. Evidence of bimanual coordination training effect on spontaneous transition frequency

As a pre-requisite, we analyzed the effects of bimanual training to resist to the transition from the AP to the IP pattern on changes in spontaneous transition frequency and the number of transitions. Results showed that repetitive bimanual coordination practice at high frequencies delayed spontaneous transition frequency of about 0.5 Hz. This result extend those observed by Temprado et al. (2002), in young adults, by showing that a reserve of behavioral flexibility still persisted in older adults, which allowed improving motor adaptability thanks to an appropriate training protocol.

Presumably, training to maintain the AP pattern was hypothesized to improve inhibition mechanisms. Accordingly, an important issue was whether the effects of bimanual coordination training on underlying inhibition mechanisms transferred to untrained motor task (i.e., intentional switching) and cognitive tasks (CWST and MAPIT) that involved, at least in part, similar cognitive mechanisms. The subsequent analyzes performed on the different dependent variables allowed to test this hypothesis.

### 4.2. Transfer of bimanual coordination training effects to the intentional switching task

The results observed for the intentional switching task (i.e., RTs and TSTs), in the post-test and the retention test, showed a transfer of bimanual coordination training to performance in the untrained intentional switching task.

First of all, for both RTs and TSTs, a significant difference was found between the two directions of switching (AP to IP and IP to AP), independent of the assessment session (pre-test, post-test and retention test). Specifically, analyses of RTs revealed that it was more difficult to dismantle the AP pattern to switch to the IP, while analyses of TSTs revealed it was more difficult to stabilize the AP pattern when switching from the IP pattern. These results are consistent with the hypothesis that, in older adults, dismantling and re-stabilizing the AP pattern more strongly involved inhibitory functions (i.e., more cognitive load) than switching from the IP pattern or stabilizing it after switching from the AP pattern.

Regarding the effects of training, as expected, both RTs and TSTs significantly decreased during post-test and retention test, in both directions of switching. Due to the required the suppression of a concurrent response (the IP pattern) to maintain the AP pattern in the training task, these results strongly suggest that inhibition processes were involved in maintaining the AP at higher frequencies during bimanual training. In this respect, they extend those reported by Temprado et al. (2002) in younger adults by showing that, in older adults, inhibitory processes were involved to resist to the spontaneous transition from AP to IP, in addition to attentional mechanisms.

Accordingly, the results observed in the pre-test and the retention test in the intentional switching task suggested training-related improvements of transition frequency presumably reflected enhanced efficiency of inhibition processes, which finally transferred to the untrained motor tasks involving (at least in part) similar cognitive mechanisms to facilitate the production of a new response by inhibiting the current one. This hypothesis was confirmed by the results observed in the CSWT and the MAPIT.

### 4.3. Transfer to the CWST and the MAPIT

In the CWST, RTs decreased in post-training sessions in both congruent and incongruent conditions. These results suggest that improvements in inhibitory mechanisms resulting from bimanual coordination training also transferred to an untrained cognitive task, at least in the incongruent condition, which involves similar mechanisms. Morevoer, the lack of interaction between time and conditions in the Stroop task suggests that bimanual coordination training also improved other cognitive functions (e.g., processing speed, attention…), which are presumably involved in the CWST (for a consistent interpretation, see mediation analyses). These findings are consistent with those reported by Temprado et al. (2020), which showed that, in older adults, inhibition mechanisms assessed through the CSWT, mediated performance in the intentional switching task, at least for the AP to IP direction.

The MAPIT was used to distinguish possible separate effects of training on motor and perceptual inhibition. Indeed, it has been shown that bimanual coordination not only results from the prevalence of neuromuscular constraints (i.e., simultaneous activation or homologous/non-homologous muscle groups) [e.g., (Kelso, 1984)], but also from perceptual (in particular, visual) constraints [e.g., (Zaal et al., 2000; Mechsner et al., 2001)], though to a lesser extent (Salter et al., 2004). Results showed a reduction in MI interference following training, but not on the PI interference score. These findings confirm that MAPIT is suitable to assess the involvement of inhibition mechanisms in cyclic, bimanual movement tasks, which has been a matter of debate in the literature [e.g., (Hervault et al., 2019)]. Secondly, they suggest that bimanual coordination training improved motor inhibition mechanisms instead of perceptual ones, which is consistent with the predominance of neuromuscular constraints in bimanual coordination dynamics (Kelso, 1984). In addition, this result suggests that perceptual and motor inhibition are separate mechanisms, not necessarily related in bimanual coordination performance (but see Netz et al., 2023, for different results).

Mediation analyses allowed us to further explore the role of inhibition mechanisms in the transfer effects of bimanual training. Taken together, these analyses confirmed: (i) the mediation by inhibition mechanisms assessed with the CWST in the improvement of responses times in the intentional switching task, especially in the AP to IP direction and (ii) the mediation by (inhibition) mechanisms involved in the intentional switching in the improvement of CWST performance, especially in the incongruent condition. These findings confirm that inhibition functions are strongly involved in maintaining and, therefore, dismantling and re-stabilizing the AP pattern. It could explain why intentional switching was longer from AP to IP than from IP to AP in the present study (see Temprado et al., 2020, for confirming evidence and a convergent interpretation). Notably, the PI and MI inhibition mechanisms were not involved in any mediating effects. Also, with respect to the CSWT, the lack of time x condition interaction was rather unexpected. It suggests that, at least, information processing speed has been improved, together with other processes involved to perform the task (i.e., inhibition). However, according to the fact that: (i) the to-be-trained task strongly loaded inhibition mechanisms and (ii) mediation analyzes was only significant for the incongruent RT, we contend that inhibition processes were sensitive to the transfer effect.

The question remains, however, of the different brain structures and mechanisms that could contribute to the observed effects. Identifying the neural underpinnings of training transfer effects might be an objective for future studies. With respect to the underlying mechanisms, interestingly, in the present study, learning and transfer effects were observed following a short-duration training (50 trials), compared to that used by Voelcker-Rehage et al. (2011). These results suggest that the benefits of “guidance” obtained thanks to this short-duration training could be based on other mechanisms than the “facilitation” effects resulting from the release of neurotrophic factors, during the training of longer duration.

## 5. Conclusion

The present study contributes, in different ways, to the existing literature on exercise and cognition in older adults. First of all, it confirmed that complex motor skills training may allow exploiting the flexibility reserve that persists in the aging neuro-cognitive system to enlarge behavioral adaptability facing higher levels of task constraints. In this respect, our results are consistent with the existing literature demonstrating the positive impact of whole-body coordination training (Voelcker-Rehage et al., 2011) or motor fitness (Voelcker-Rehage et al., 2010) on cognitive functions. They show that bimanual coordination is a suitable task to achieve this objective. Indeed, in the present study, bimanual coordination training allowed effectively and quickly improving cognitive mechanisms in non-practiced cognitive and motor tasks. Thus, it can be concluded that when common cognitive mechanisms are at work in cognitive and motor tasks, training them in motor tasks may transfer to cognitive tasks. It remains however to determine whether the present finings can be extended to other types of complex movements.

Thirdly, this study is the first to test and detect positive cognitive improvements after a bimanual coordination training, which is of high impact for the field of age-related cognitive-decline prevention. Indeed, our findings (re)open the question of what type of physical and/or motor training programs may be most effective to improve cognition and cognitive-motor behavior in older adults (see also (Raichlen and Alexander, 2017; Tait et al., 2017; Herold et al., 2018; Torre et al., 2021; Torre and Temprado, 2022a,b). Indeed, though it is commonly considered that endurance training should be preferably used in combination with cognitive stimulations (Torre et al., 2021; Torre and Temprado, 2022a,b), when referring to the program duration, our results suggest that complex motor skill training might be even more efficient than endurance training for obtaining strong and transferable effects on cognitive function. Thus, instead of combining physical (endurance) training and cognitive stimulations, a promising combination might be those associating endurance training and complex motor skills training. This hypothesis has been scarcely been addressed in the literature (Raichlen and Alexander, 2017) and should be the objective of further studies. In particular, the present results remain to be extended to different complex motor skills training programs.

## 6. Limitations of the study

A limitation of the present study might be the lack of a control group. Indeed, while transfer effects of the training were strong and reliable they could, at least in part, reflect a test-retest effect. Notably, few studies showed that Stroop task delivered repeteadly through smartphone application may be used as a short and valid method to screen some forms of encephalopathy, thereby suggesting that it the Stroop test presents a high test/re-test reliability and is resistant to test/re-test learning effect (see Franzen et al., 1987 for confirming evidence). In addition, the causal effect of the intervention on cognition was explored through mediation analyses, which allowed quantifying the extent to which a variable participates in the transmittance of change from a cause to its effect. Specifically, mediation analyses explored how the changes in bimanual coordination from before to after the training, participated in the improvement in cognitive abilities. While it does not replace control group, mediation analyses give a robust understanding of the relationship between cognition and bimanual coordination, and how training coordination could improve cognitive abilities. In this respect, these analyses suggested that the presence of a test-retest effect was not enough to explain the observed results. One could argue that test retests effects may be more pronounced in some participants and not others, causing the within subject association between mediators changes and outcomes changes. While this could explain some of the results, mediation were only seen in a small specific part of our outcomes (not known to be more sensitive to test retests than the others). Distinguishing fast from slow learners, if the formers, in the mediator fields are the same in the outcome field, and given the high specificity of these variables among our set of variables, this highlights a strong relationship between mediator and outcomes. Nevertheless, further studies are necessary to confirm these results and to extend them to different complex motor skills training programs.

## Data availability statement

The raw data supporting the conclusions of this article will be made available by the authors, without undue reservation.

## Ethics statement

The studies involving human participants were reviewed and approved by CERSTAPS IRB00012476-2022-12-05-181. The patients/participants provided their written informed consent to participate in this study.

## Author contributions

All authors contributed to the study's conception and design. Material preparation, data collection, and analysis were performed by MT and LA. The first draft of the manuscript was written by MT and revised by J-JT and AL. All authors commented on previous versions of the manuscript. All authors read and approved the final manuscript.
